# Effects of Exercise on DNA Methylation: A Systematic Review of Randomized Controlled Trials

**DOI:** 10.1007/s40279-024-02033-0

**Published:** 2024-06-05

**Authors:** Paula Etayo-Urtasun, Mikel L. Sáez de Asteasu, Mikel Izquierdo

**Affiliations:** 1 Navarrabiomed, Pamplona, Spain; 2 Department of Health Sciences, Hospital Universitario de Navarra (HUN)-Universidad Pública de Navarra (UPNA), IdiSNA, Av. De Barañain s/n, 31008 Pamplona, Navarra Spain; 3 CIBER of Frailty and Healthy Aging (CIBERFES), Instituto de Salud Carlos III, Madrid, Spain

## Abstract

**Background:**

Regular exercise reduces chronic disease risk and extends a healthy lifespan, but the underlying molecular mechanisms remain unclear. DNA methylation is implicated in this process, potentially altering gene expression without changing DNA sequence. However, previous findings appear partly contradictory.

**Objective:**

This review aimed to elucidate exercise effects on DNA methylation patterns.

**Methods:**

PubMed, Scopus and Web of Science databases were searched following PRISMA 2020 guidelines. All articles published up to November 2023 were considered for inclusion and assessed for eligibility using the PICOS (Population, Intervention, Comparison, Outcomes and Study) framework. Randomized controlled trials that assessed the impact of exercise interventions on DNA methylation in previously inactive adults were included. We evaluated the methodological quality of trials using the PEDro scale.

**Results:**

A total of 852 results were identified, of which 12 articles met the inclusion criteria. A total of 827 subjects were included in the studies. Intervention lengths varied from 6 weeks to 12 months. Most trials indicated that exercise interventions can significantly alter the DNA methylation of specific genes and global DNA methylation patterns.

**Conclusions:**

The heterogeneity of results may arise from differences in participant demographics, intervention factors, measurement techniques, and the genomic contexts examined. Future research should analyze the influences of activity type, intensity, and duration, as well as the physical fitness outcomes on DNA methylation. Characterizing such dose–response relationships and identifying genes responsive to exercise are crucial for understanding the molecular mechanisms of exercise, unlocking its full potential for disease prevention and treatment.

## Key Points

**Table:** 

Exercise interventions can induce significant modifications in DNA methylation, including a downregulation of global DNA methylation and an upregulation of DNA methylation levels associated with key genes such as RANKL, FKBP5, AURKA, BPIFA, BRCA1, p66Shc, and ASC.
While determining an exact effective dose for inducing significant changes in DNA methylation remains challenging, evidence suggests that prescribed workloads must enhance fitness levels by combining aerobic exercise and strength training to successfully alter DNA methylation.

## Introduction

A growing body of scientific evidence indicates that regular exercise can reduce the risk of developing several diseases or conditions, including obesity, cancer, depression, type 2 diabetes, and cardiovascular diseases [1]. Furthermore, a recent meta-analysis with data from 1.44 million individuals suggested that exercise can decrease the risk of several types of cancer, including breast, colon, endometrial, head and neck, esophageal, lung, kidney, blood, and bladder cancers [2]. In the meta-analysis by Posadzki et al. [3], which included 27,671 participants, exercise was associated with a 13% reduction in mortality rate.

While the benefits of exercise have been documented extensively, the underlying molecular mechanisms driving exercise-induced adaptations remain largely elusive [4–6]. One promising hypothesis posits that an exercise intervention may reduce disease risk through inducing epigenetic modifications [7]. These epigenetic modifications alter chromatin structure and consequently modulate transcriptional accessibility without changes to the nucleotide sequence itself [4, 8, 9].

DNA methylation, one of the most studied epigenetic markers, involves the transfer of a methyl group to the fifth carbon of the cytosine-pyrimidine ring, forming 5mC [4, 10–15]. This process primarily occurs at CpG sites, which are specific regions in the DNA sequence where cytosine and guanine nucleotides are connected by a phosphate group. DNA methylation plays a vital role in maintaining chromosomal stability and regulating gene expression [12, 16].

DNA methylation plays a crucial a role in the onset and progression of various diseases. Firstly, altered DNA methylation has been associated with several autoimmune diseases, including rheumatoid arthritis, systemic lupus erythematosus, and multiple sclerosis [17]. Secondly, it plays a part in regulating biological processes related to cardiovascular diseases, such as atherosclerosis, hypertension, and inflammation [18, 19]. It is also a significant risk factor for metabolic disorders such as type 2 diabetes [20]. Additionally, DNA methylation is closely associated with neurological diseases, with Alzheimer's disease and Parkinson's disease being two extensively studied examples [21]. Dysregulated DNA methylation is also known to be involved in cancer initiation and progression. Hypomethylation in repetitive sequences and gene bodies, combined with hypermethylation in the promoter regions of tumor suppressor genes, leads to abnormal gene transcription. This contributes to increased chromosomal translocations and gene mutations that contribute to cancer development [12].

Several studies have reported that exercise is associated with changes in DNA methylation patterns across the human genome, thus modifying gene expression patterns in multiple tissues [10, 22]. However, much research establishing this relationship has been conducted in animals and findings may not generalize to humans [23, 24]. Regarding human studies, most have utilized cross-sectional designs or clinical trials without control groups. Consequently, existing reviews on this topic have included studies with promising but non-causal results [7, 8, 10].

Randomized controlled trials carried out thus far have shown contradictory results. For example, Boyne et al. [25] found no significant DNA methylation differences in repetitive sequences and specific genes following a 12-month aerobic exercise intervention. However, an equal-duration intervention involving supervised Nordic walking effectively induced significant restoration of p66Shc gene promoter methylation [26].

To address these inconsistencies, a systematic review and synthesis of evidence on exercise effects on DNA methylation is crucial. The primary aim of this review was to consolidate high-quality studies that analyze the impact of exercise interventions on DNA methylation. Given DNA methylation´s role in gene expression and transcription regulation, characterizing exercise-induced epigenetic modifications is a critical step in uncovering the molecular mechanisms that underpin the well-documented disease prevention benefits of physical activity. Understanding these mechanisms is vital for developing tailored exercise prescriptions for health optimization.

## Methods

### Study Design

This systematic review included studies that analyzed the effects of exercise interventions on DNA methylation. It was conducted following the guidelines summarized in the PRISMA (Preferred Reporting Items for Systematic Reviews and Meta-Analyses) statement [27].

### Search Strategy

The end date of the search for studies was 17 November 2023. Articles were collected from the following databases: Medline (PubMed), Web of Science, Cochrane, and Scopus. We applied a search filter to retrieve only studies published from 2013 onward. In order to conduct the search, we applied the following combination of descriptors to the title in all the aforementioned databases: (methylation OR epigenetic) AND (exercise OR "physical activity" OR training).

### Eligibility Criteria

Inclusion criteria were defined using the PICOS (Population, Intervention, Comparison, Outcomes and Study) model [28]. Articles were included if they met the following criteria: (a) included sedentary and/or physically inactive adults over 18 years of age, (b) evaluated the effects of an exercise intervention, (c) included a control group or a group with different training modalities, (d) evaluated DNA methylation, and (e) were randomized controlled trials.

Trials were excluded if the participants were children. Moreover, those studies that included dietary modifications were also excluded, since they did not allow evaluation of the exercise-induced effects in isolation. We did not include reviews, observational studies, or clinical trials without a control group. Thus, after examining the inclusion and exclusion criteria, we selected a total of 12 randomized controlled trials for this systematic review.

### Data Collection and Selection Process

The search strategy consisted of combining all the results from the four databases and eliminating duplicates. Articles identified were evaluated by reading their title and abstract. A full reading of the selected articles was then performed and articles that did not meet the inclusion criteria were excluded.

Data extraction was carried out by the main author (PEU) regarding study information (i.e., author name, publication year, enrollment setting, and sample size), population (i.e., age, sex), exercise characteristics (i.e., duration, type of activity, frequency as sessions/week, volume as min/session and sets and repetitions/session, intensity), and outcomes (i.e., tissue, DNA methylation measurement, and analyzed genes).

### Evaluation of Methodological Quality

We evaluated the methodological quality and assessed the risk of bias for included randomized controlled trials using the validated Physiotherapy Evidence Database (PEDro) rating scale (Table 1). According to Maher et al. [29], the PEDro scale is a reliable 11-item scale designed to measure the methodological quality of randomized controlled trials. The score obtained is qualified as high when the value ranges between 6 and 10 (the first item does not compute for the total score). The methodological quality of studies with a score between 4 and 5 points is considered moderate, and scores of 3 or less indicate low quality.

**Table 1:** Evaluation of methodological quality using the PEDro (Physiotherapy Evidence Database) scale

Study	1	2	3	4	5	6	7	8	9	10	11	Scoreª	Quality
Chelly et al. [30]	Y	Y	Y	N	N	N	N	Y	Y	Y	Y	6	High
Willmer et al. [31]	Y	Y	Y	Y	N	N	N	N	Y	Y	Y	6	High
Duggan et al. [13]	Y	Y	Y	Y	N	N	Y	Y	Y	Y	Y	8	High
Gillman et al. [15]	Y	Y	Y	Y	N	N	N	N	Y	Y	Y	6	High
Streese et al. [26]	Y	Y	Y	Y	N	N	Y	N	Y	Y	Y	7	High
Boyne et al. [25]	Y	Y	Y	Y	N	N	N	N	Y	Y	Y	6	High
Butts et al. [32]	Y	Y	Y	N	N	N	N	Y	Y	Y	Y	6	High
Dimauro et al. [33]	Y	Y	Y	Y	N	N	N	Y	Y	Y	Y	7	High
Bryan et al. [34]	Y	Y	Y	Y	N	N	N	N	Y	Y	Y	6	High
Denham et al. [35]	Y	Y	Y	N	N	N	N	Y	Y	Y	Y	6	High
Ngwa et al. [36]	Y	Y	Y	N	N	N	Y	Y	Y	Y	Y	7	High
Hwang et al. [37]	Y	Y	Y	Y	N	N	N	Y	Y	Y	Y	7	High

*N* no; *Y* yes

^a^From a possible maximal score of 10. 1: Eligibility criteria were defined. 2: Subjects were randomly assigned to groups. 3: Allocation was concealed. 4: Major prognostic indicators were similar between groups at baseline. 5: All subjects were blinded. 6: All therapists/researchers who administered the therapy/protocol were blinded. 7: There was blinding of all evaluators who measured at least one key outcome. 8: Measures of at least one key outcome were obtained from more than 85% of subjects who were initially assigned to groups. 9: All subjects for whom outcome measures were available received the assigned treatment or control condition. 10: Results of statistical comparisons between groups were reported for at least one key outcome. 11: The study provided point measures and measures of variability for at least one key outcome

## Results

### Study Selection

**Fig. 1:**
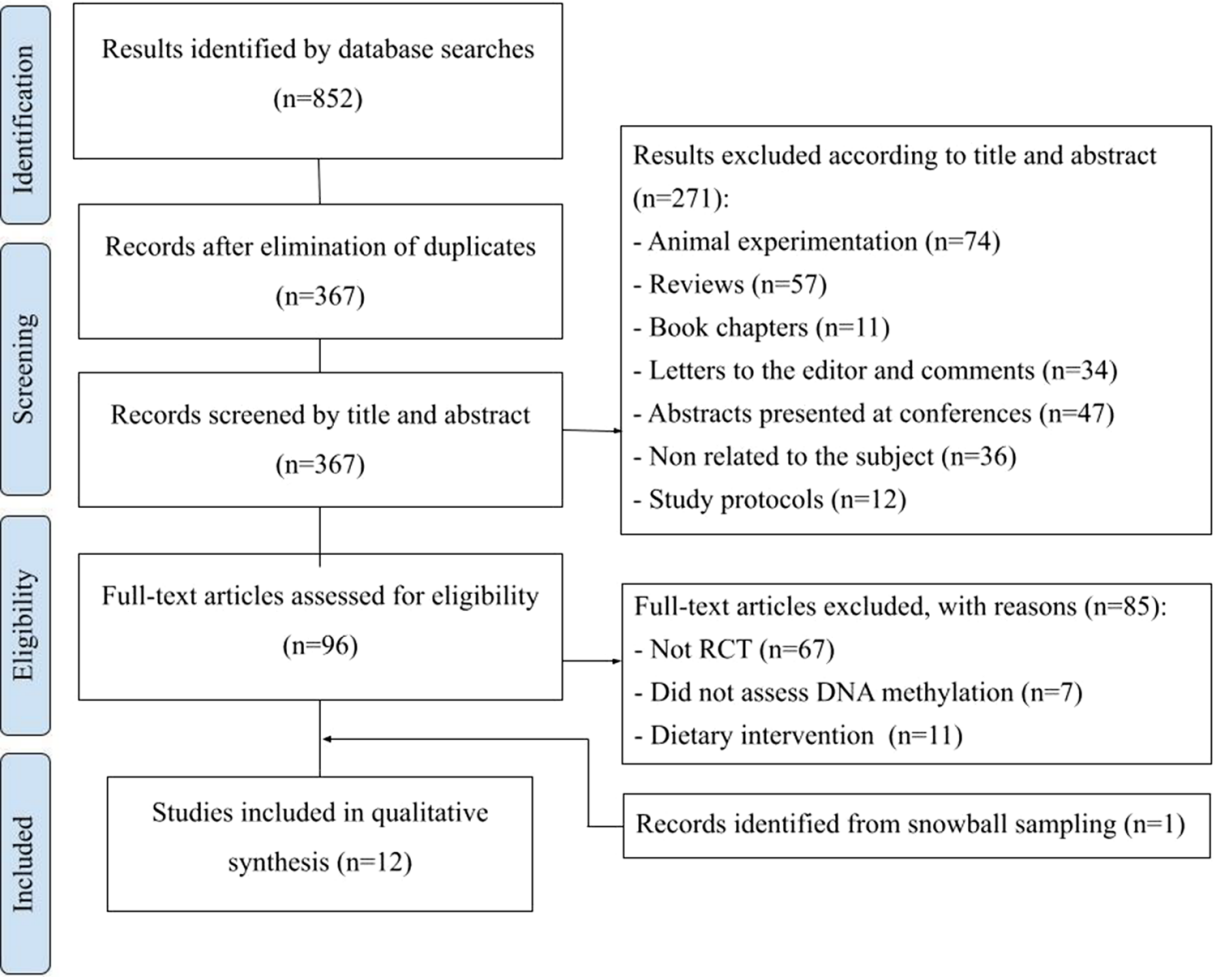
PRISMA flow diagram of the screening process. *N* number of studies, *RCT* randomized controlled trials

Figure 1 shows a flow diagram detailing the literature search process. The database search yielded 852 total studies, with the most results obtained from Web of Science (*n* = 336). Similar numbers of articles were found in Scopus (*n* = 249) and Medline (PubMed) (*n* = 220). In Cochrane, there was a lower number of results (*n* = 47). Following the removal of 485 duplicate articles, 367 studies remained. Screening of titles and abstracts led to the exclusion of 271 articles based on pre-defined criteria. Of the 96 full-text articles evaluated for eligibility, 85 were excluded for not meeting all inclusion criteria. Checking the references of relevant articles identified one additional eligible study. In total, 12 studies met the criteria and were incorporated into the qualitative synthesis.

### Characteristics of Included Studies

The 12 included trials comprised 827 total participants who were sedentary or physically inactive prior to the interventions. Most participants were healthy adults; however, some studies included clinical populations such as those with mild cognitive impairment [36], stable heart failure [32], colorectal cancer [37], obesity [31], or increased cardiovascular risk [26]. Participants tended to be middle-aged adults (mean age ~ 55 years), apart from one young adult sample [15]. Three studies enrolled only women [15, 25, 31] and one only enrolled men [35], while the remainder analyzed mixed-sex samples [13, 26, 30, 32–34, 36, 37]. Overall, the dropout rates were relatively moderate, although variations were observed across the studies. Some studies have reported a high level of adherence, with over 85% of participants completing the trial [13, 25, 26]. In contrast, other studies have reported a higher dropout rate, with completion percentages ranging from 60 to 70% [15, 31].

Most interventions utilized supervised exercise training [15, 26, 30, 31, 33, 35]. Some interventions combined supervised and unsupervised sessions [13, 25, 36], while one study provided only a single supervised session [37]. Two studies provided personalized counseling without direct supervision [32, 34]. The most common exercise modality was aerobic training, although some programs implemented resistance training. Intervention length ranged from 6 weeks to 12 months, with 12 weeks being most common. Session duration ranged from 15–20 min to 45–60 min, performed 3–5 days per week at intensities between 50 and 85% maximal heart rate. Control groups typically maintained usual lifestyle without adding exercise. Some control groups performed stretching [32] or received standard physical activity advice [34].

All studies evaluated exercise-induced changes in DNA methylation using either bisulfite-based (e.g., pyrosequencing) [15, 25, 30–32] or microarray-based methods (e.g., Illumina arrays) [34, 36, 37]. Most examined gene-specific methylation [13, 15, 25–32, 34], while some analyzed genome-wide [35–37] or global methylation [33–35]. Four studies found significant between-group differences in targeted genes [26, 30–32], while two did not [13, 25]. Two additional studies found differences only for certain examined genes [15, 34]. All three studies analyzing global methylation observed significant exercise-induced reductions [33–35]. The epigenome-wide analyses each identified numerous differentially methylated genes post-intervention [35–37]. Table 2 summarizes the key characteristics and findings of all included studies.

**Table 2:** Qualitative analysis of randomized controlled trials

Study	Year	Participants	Intervention	Tissue	DNA measurement	Analyzed genes	Outcome (between-group comparisons)
Chelly et al. [30]	2023	52 subjects aged 24–50 years (13 EG and 11 CG)	EG: 3 months of supervised walking, resistance and stretching exercises, 4 times/week at 60% THR CG: usual lifestyle	Peripheral blood	Quantitative Methylation-Specific PCR (qMSP)	RANKL	DNA methylation was significantly increased at the RANKL gene promoter in EG compared to CG (*p* = 0.0005)
Willmer et al. [31]	2022	31 women with obesity aged 20–35 years (19 EG and 12 CG)	EG: 12 weeks of supervised aerobic/resistance training, 4 times/week at 60–70% MHR for 40–60 min CG: usual lifestyle	ASAT and GSAT	Bisulfite Pyrosequencing	FKBP5	DNA methylation was significantly increased at the FKBP5 gene in EG compared to CG (*p* = 0.019 in GSAT, *p* = 0.03 in ASAT)
Duggan et al. [13]	2022	202 subjects aged 40–75 years (100 EG and 102 CG)	EG: 12 months of aerobic exercise, 6 times/week (3 of them supervised and the remainder at home) at 60–85% MHR for 60 min CG: usual lifestyle	Colon tissue	Droplet Digital Methylation Specific PCR (MethyLight ddPCR)	ESR1, EVL and p14ARF	No significant between-group differences were found in DNA methylation at p14ARF (*p* = 0.82), EVL (*p* = 0.29) or ESR1 (*p* = 0.12)
Gillman et al. [15]	2021	135 women aged 30–45 years (33 EG1, 31 EG2, 41 EG3 and 32 EG4)	16 weeks of supervised aerobic exercise 4 times/week EG1: 40 min high intensity EG2: 20 min high intensity EG3: 40 min moderate intensity EG4: 20 min moderate intensity	Peripheral blood	Pyrosequencing	BRCA1, RUNX3, SIM1, AURKA, BCAR1, BPIFA4P, GALNT9, FBLN2, PAX6, TLR4 and TLR6	DNA methylation was significantly increased in EG compared to CG at the following genes: AURKA: *p* = 0.04 BCAR1: *p* = 0.06 BPIFA4P: *p* = 0.005 BRCA1: *p* = 0.04 No significant between-group differences were found in SIM1, FBLN2, GALNT9, PAX6, RUNX3, TLR4 and TLR6
Streese et al. [26]	2020	84 subjects with ≥ 2 CV risk factors aged 50–80 years (44 EG and 40 CG)	EG: 12-week Nordic walking-based and supervised HIIT 3 times/week at 75–90% MHR CG: PA recommendations	Peripheral blood	Real-time quantitative PCR amplification	p66^Shc^	DNA methylation was significantly increased at the p66^Shc^ gene promoter in EG compared to CG (*p* = 0.034)
Boyne et al. [25]	2018	320 women aged 50–75 years (160 EG and 160 CG)	EG: 12 months of progressive aerobic exercise. 3–5 times/week at 50–80% MHR for 45 min CG: usual lifestyle	Peripheral blood	Pyrosequencing	LINE-1, Alu, APC, BRCA1, RASSF1 and hTERT	No significant between-group differences were found in DNA methylation of LINE-1 and Alu repeats or at the APC, BRCA1, RASSF1 and hTERT gene promoters (*p* ≥ 0.05)
Butts et al. [32]	2018	54 stable outpatients with heart failure aged 40–75 years (38 EG and 16 CG)	EG: 12 weeks of progressive aerobic training. Walking 3 times/week at 60–70% MHR for 30–45 min CG: stretching exercises	Peripheral blood	Pyrosequencing	ASC	DNA methylation was significantly increased at the ASC gene promoter in EG compared to CG (*p* = 0.006)
Bryan et al. [34]	2013	64 healthy subjects aged 18–45 years (37 EG and 27 CG)	EG: 12 months of individually tailored mails containing personalized PA counseling CG: standard self-help material	Saliva	Illumina Infinium Human Methylation27 BeadChip assay	Global DNA methylation	No significant between-group differences were found in global DNA methylation (*p* = 0.99). Increasing PA minutes significantly reduced global DNA methylation (*p* = 0.04)
Dimauro et al. [33]	2016	20 subjects aged 70–75 year (10 EG and 10 CG)	EG: 12 weeks of explosive-type training 2 times/week. 3–4 sets of 10–12 repetitions at 70% of 1RM CG: usual lifestyle	Peripheral blood	Methylated DNA Quantification Kit MDQ1	Global DNA methylation	Global DNA methylation was significantly decreased in EG compared to CG (*p* ≤ 0.05)
Denham et al. [35]	2015	24 men aged 18–30 years (13 EG and 11 EG)	EG: 12 weeks of sprint interval training 2 times/week CG: usual lifestyle	Sperm	5-mC ELISA Kit and Infinium Human-Methylation 450 K BeadChip	Global and genome-wide DNA methylation	Global DNA methylation was significantly reduced in EG compared to CG (*p* = 0.006). Most changed 10 CpG sites corresponded to genes WBSCR17, SEL1L, SLC25A36, CBX5, C1orf53, RAMP1, FNBP1, CACNA1A, DNAJB7 and XPNPEP3
Ngwa et al. [36]	2022	19 mild cognitively impaired elderly subjects aged > 55 years (11 EG and 8 CG)	EG: 6 months of supervised progressive aerobic training at 50–70% VO_2_max 3 times/week for 20–40 min and an unsupervised walking session for 45–60 min at lower intensity CG: stretching exercises	Peripheral blood	Illumina Infinium HumanMethylation450 BeadChip	Genome-wide DNA methylation	214 CpG sites were significantly hypomethylated and 34 hypermethylated in EG compared to CG. The top CpG sites corresponded to genes VPS52, SCARB1, ARTN, NR1H2, PPP2R5D, CAB39, SNORD115-41 and DLL4
Hwang et al. [37]	2022	15 colorectal cancer survivors aged > 19 years (9 EG and 6 CG)	EG: 6 weeks of 6 home-based exercise sessions and one supervised session containing resistance and stretching exercises CG: usual lifestyle	Peripheral blood	Infinium Methylation-EPIC BeadChip Kit	Genome-wide DNA methylation	756 CpGs within 524 genes showed a significant change in EG compared to CG. The top CpG sites corresponded to genes STK38L, NRXN1, FOXD3, LTBP1, HTR3A, UBE3C, ARG1, CHN2 and CH13L1

*ASAT* abdominal subcutaneous adipose tissue, *CG* control group, *CV* cardiovascular, *EG* exercise group, *GSAT* gluteal subcutaneous adipose tissue, *HIIT* high-intensity interval training, *MHR* maximal heart rate, *PA* physical activity, *RM* repetition maximum, *THR* target heart rate

## Discussion

Most included studies demonstrated that exercise interventions can induce significant changes in DNA methylation. Trials evaluating specific CpG sites observed increased methylation related to RANKL, FKBP5, AURKA, BPIFA, BRCA1, p66^Shc^, and ASC genes. Regarding global methylation, studies showed significant downregulation resulting from exercise. Similarly, epigenome-wide analyses revealed exercise alters the DNA methylation profile. The vast majority of trials are in line with previous cross-sectional studies reporting significant effects of exercise on DNA methylation [7, 8, 10].

Several factors may explain the differences between findings. First, demographics such as age, sex, and ethnicity can drive heterogeneity [7, 8, 38]. Second, intervention factors including duration and intensity seem key, as 6–12 months may suffice to alter age-related genes [7], while optimal intensity remains unclear [10]. The type of exercise performed during an intervention may also be a key factor influencing epigenetic modifications. Significant changes in DNA methylation included both aerobic exercise and strength training [30, 31]. In contrast, in a study by Duggan et al. [13], participants only performed aerobic exercise training without strength training. Although the intervention lasted 12 months with a frequency of six sessions per week at moderate to vigorous intensity, no significant changes in DNA methylation were observed, suggesting that including strength training may be crucial. Similarly, in the study by Boyne et al. [25], no significant changes in DNA methylation were observed despite a duration of 12 months, frequency of three to five times per week, and moderate to vigorous intensity. Taken together, these results indicate the importance of strength training in driving changes in DNA methylation.

In addition, most studies that reported significant differences in DNA methylation also documented improvements in physical fitness. For example, in a study by Chelly et al. [30], participants in the training group showed increased DNA methylation of the RANKL gene along with enhanced maximal oxygen consumption (VO_2_max). Willmer et al. [31] observed significant DNA methylation changes that were accompanied by an improvement in cardiorespiratory fitness, measured as peak oxygen consumption (VO_2_peak). In the study by Boyne et al. [25], after correlating changes in DNA methylation with improvements in physical fitness (measured as VO_2_max), a significant dose–response relationship emerged between physical fitness and RASSF1 methylation in a group of inactive postmenopausal women. Hence, despite the constraints of this study suggesting that the findings should be viewed as preliminary, it seems that improved physical fitness could be a critical factor in the influence of exercise on DNA methylation.

The primary outcome of this study might account for the observed differences in results, suggesting that gene-specific effects of exercise could contribute to inconsistencies. While gene-specific studies enable hypothesis testing, observed differences may be attributable to differential responsiveness across various genes [8, 13]. For instance, Duggan et al. [13] found no significant differences in DNA methylation between groups for specific genes such as p14ARF, EVL, and ESR1. This result does not negate the possibility that exercise interventions may lead to significant DNA methylation changes in other genes, but rather indicates no observed changes in the genes under examination when compared to the control group. Most gene-focused studies have reported an increase in DNA methylation following exercise interventions [15, 26, 30–32]. However, studies on global DNA methylation have frequently reported a significant decrease [33–35]. Although this appears contradictory, it may not be, as changes in global DNA methylation reflect changes that likely encompass numerous genes, some undergoing hypermethylation and others hypomethylation. Consequently, global assessments may miss the subtleties of gene-specific effects [10]. Epigenome-wide association studies (EWAS) have identified significant variations in specific genes between exercise and control groups, but these findings include the risk of false positives due to the exploratory nature of such studies, potentially mistaking unrelated changes for effects of the intervention [34].

DNA methylation assessments also differed in tissue type. As patterns change across tissues [7, 10, 11], contrasting findings could reflect this specificity. For instance, Duggan et al. [13] found no DNA methylation changes in colon tissue, while Denham et al. [15] observed reduced sperm DNA methylation after exercise training. In addition, both studies analyzed different genes, which may indicate that exercise has effects on specific tissues and genes.

Moreover, methods used to quantify methylation differ. Pyrosequencing enables simple, rapid, and reliable analysis [39], but microarrays have higher genomic coverage [34, 35]. Within platforms, designs target varying genomic regions with different methylation statuses [8].

Reduced DNA methylation found in some studies aligns with increased DNA methylation in others when considering regulatory effects on transcription [26, 34]. Promoter methylation is associated with silencing and loss therefore enhances expression [4, 8, 10]. However, gene body methylation can increase expression, explaining the hypomethylation observed by Ngwa et al. [36] within VPS527.

### Limitations

This systematic review has limitations including small sample sizes [13, 35] and predominant use of heterogeneous blood samples without cell type control [8, 15, 25]. Differing methods and tissues also complicate comparisons. While our methodological approach was in strict accordance with PRISMA 2020 guidelines and employed the PEDro scale to enhance the review's reliability and robustness, we must acknowledge certain limitations inherent to these methodologies. A significant limitation arises from the heterogeneity among the included studies, with variability in participant characteristics, intervention protocols, measurement methods, and outcome measures possibly affecting the heterogeneity and, consequently, the review's overarching conclusions. Additionally, despite meticulous data selection and extraction processes aimed at mitigating bias, the potential for inherent biases in study selection and interpretation persists. These limitations need to be considered when interpreting the review's findings, and subsequent research should strive to tackle these challenges, thereby reinforcing the evidence base in this domain. However, the review uniquely summarizes exercise intervention effects on DNA methylation using only randomized controlled trials [15], further controlling bias through inclusion of sedentary, non-smoking samples.

Though epigenetic exercise research is nascent, findings could enable use of DNA methylation to track intervention impacts on disease risk. This could motivate adherence by demonstrating behavioral change effects. However, practical applications remain speculative.

Future investigations should consider several factors. First, studies should evaluate DNA methylation based on demographics such as age and ethnicity, which can influence patterns [7]. Second, specific tissue- and cell-specificity impacts should be examined across bodily tissues, controlling for cell type distributions in blood [8]. This could reveal differentially susceptible genomic regions. While global assessments have utility, gene-specific analyses are essential for elucidating disease pathways [10]. Identifying exercise-responsive genes represents a key step for assessing epigenetic links between exercise and pathology. Additionally, comparing activity types, intensities, and durations in clinical trials is needed to elucidate dose–response relationships. Considering fitness impacts may also be valuable, as evidence suggests fitness strongly predicts DNA methylation changes [15, 38]. Analyzing potential interactions with diet and mediation by body composition represents other avenues worth exploring. Ultimately, besides randomized controlled trials, it would also be worthwhile conducting longitudinal studies, which may offer a more comprehensive understanding of the temporal dynamics of exercise-induced DNA methylation changes.

In summary, our analysis highlights the intricate relationship between exercise, DNA methylation, and prevention of chronic diseases. By identifying the key genes and genomic profiles responsive to exercise interventions, we pave the way for personalized approaches to health promotion and disease management. The insights gained from this study not only contribute to advancing our understanding of the molecular mechanisms underlying the benefits of exercise, but also hold promise for developing targeted interventions that harness the full therapeutic potential of physical activity. Continued exploration of dose–response relationships and comprehensive analysis of exercise parameters will be crucial to optimizing health outcomes and shaping public health strategies for the future. Furthermore, the identification of differentially methylated genes and pathways responsive to exercise interventions could enable the use of epigenetic biomarkers to monitor the molecular effects of exercise programs. Characterizing such profiles represents an essential step towards elucidating the mechanism of exercise.

## Conclusions

In conclusion, this review suggests that physical exercise can markedly influence DNA methylation patterns. Changes in gene expression, which drive physiological adaptation to exercise, seem to be partially governed by epigenetic modifications that either activate or silence genes. Consequently, by modulating gene expression programs, physical activity could serve as a preventative and therapeutic intervention for various diseases, presenting significant implications for public health. Nonetheless, concrete conclusions regarding the effects of regular exercise on human epigenetics are still elusive, given the complex and relatively uncharted nature of this research field. Arising from the insights of this review, we can also propose practical recommendations regarding the influence of exercise on DNA methylation. Firstly, a combination of aerobic and strength training appears crucial in eliciting changes in DNA methylation. Secondly, enhancing physical fitness should be a focal point, given its potential association with DNA methylation alterations. These recommendations could inform future exercise protocols aimed at maximizing health benefits through epigenetic pathways.

Future work should focus on specific epigenetic markers that respond to exercise. Identifying and characterizing such biomarkers could prepare the way for interventions that utilize DNA methylation to mitigate disease risk. In addition, subsequent investigations ought to appraise the cost-effectiveness of various exercise forms and their enduring epigenetic impacts. Such analysis is crucial for guiding evidence-based policy-making and fostering enduring health strategies. Moreover, there is a call for sustained collaborative research efforts to establish the optimal exercise dosages for epigenetic modifications. The formation of international data-sharing consortia using standardized metrics will be instrumental in disentangling the intricate links between physical activity and gene regulation.

## Declarations

### Availability of data and material

The datasets analyzed in the present study can be obtained on reasonable request to the corresponding author.

## References

[1] Thompson WR, Sallis R, Joy E, Jaworski CA, Stuhr RM, Trilk JL. Exercise is medicine. Am J Lifestyle Med. 2020;14(5):511–23. 10.1177/1559827620912192. 32922236 10.1177/1559827620912192 PMC7444006

[2] Moore SC, Lee I, Weiderpass E, Campbell PT, Sampson JN, Kitahara CM, et al. Association of leisure-time physical activity with risk of 26 types of cancer in 1.44 million adults. JAMA Intern Med. 2016;176(6):816–25. 10.1001/jamainternmed.2016.1548. 27183032 10.1001/jamainternmed.2016.1548 PMC5812009

[3] Posadzki P, Pieper D, Bajpai R, Makaruk H, Könsgen N, Neuhaus AL, et al. Exercise/physical activity and health outcomes: an overview of Cochrane systematic reviews. BMC Public Health. 2020;20(1):1724. 10.1186/s12889-020-09855-3. 33198717 10.1186/s12889-020-09855-3 PMC7670795

[4] Światowy WJ, Drzewiecka H, Kliber M, Sąsiadek M, Karpiński P, Pławski A, et al. Physical activity and DNA methylation in humans. Int J Mol Sci. 2021;22(23):12989. 10.3390/ijms222312989. 34884790 10.3390/ijms222312989 PMC8657566

[5] Wang Q, Zhou W. Roles and molecular mechanisms of physical exercise in cancer prevention and treatment. J Sport Health Sci. 2021;10(2):201–10. 10.1016/j.jshs.2020.07.008. 32738520 10.1016/j.jshs.2020.07.008 PMC7987556

[6] Coffey VG, Hawley JA. The molecular bases of training adaptation. Sports Med. 2007;37:737–63. 10.2165/00007256-200737090-00001. 17722947 10.2165/00007256-200737090-00001

[7] Boyne DJ, O’Sullivan DE, Olij BF, King WD, Friedenreich CM, Brenner DR. Physical activity, global DNA methylation, and breast cancer risk: a systematic literature review and meta-analysis. Cancer Epidemiol Biomark Prev. 2018;27(11):1320–31. 10.1158/1055-9965.epi-18-0175. 10.1158/1055-9965.epi-18-0175 29991518

[8] Voisin S, Eynon N, Yan X, Bishop DJ. Exercise training and DNA methylation in humans. Acta Physiol. 2015;213(1):39–59. 10.1111/apha.12414. 10.1111/apha.12414 25345837

[9] Ling C, Rönn T. Epigenetic adaptation to regular exercise in humans. Drug Discov Today. 2014;19(7):1015–8. 10.1016/j.drudis.2014.03.006. 24632002 10.1016/j.drudis.2014.03.006

[10] Horsburgh S, Robson-Ansley P, Adams R, Smith C. Exercise and inflammation-related epigenetic modifications: focus on DNA methylation. Exerc Immunol Rev. 2015;21:26–41. 25826329

[11] Grazioli E, Dimauro I, Mercatelli N, Wang G, Pitsiladis Y, Di Luigi L, et al. Physical activity in the prevention of human diseases: role of epigenetic modifications. BMC Genom. 2017;18(S8):802. 10.1186/s12864-017-4193-5. 10.1186/s12864-017-4193-5 PMC5688489 29143608

[12] Li Y, Fan Z, Meng Y, Liu S, Zhan H. Blood-based DNA methylation signatures in cancer: A systematic review. Biochim Biophys Acta Mol Basis Dis. 2023;1869(1): 166583. 10.1016/j.bbadis.2022.166583. 36270476 10.1016/j.bbadis.2022.166583

[13] Duggan C, Yu M, Willbanks AR, Tapsoba JD, Wang CY, Grady WM, et al. Exercise effects on DNA methylation in EVL, CDKN2A (p14, ARF), and ESR1 in colon tissue from healthy men and women. Epigenetics. 2022;17(10):1070–9. 10.1080/15592294.2021.1982512. 34550860 10.1080/15592294.2021.1982512 PMC9542713

[14] Lyko F. The DNA methyltransferase family: a versatile toolkit for epigenetic regulation. Nat Rev Genet. 2018;19:81–92. 10.1038/nrg.2017.80. 29033456 10.1038/nrg.2017.80

[15] Gillman AS, Helmuth T, Koljack CE, Hutchison KE, Kohrt WM, Bryan AD. The effects of exercise duration and intensity on breast cancer-related DNA methylation: a randomized controlled trial. Cancers (Basel). 2021;13(16):4128. 10.3390/cancers13164128. 34439282 10.3390/cancers13164128 PMC8394212

[16] Flores KB, Wolschin F, Amdam GV. The role of methylation of DNA in environmental adaptation. Integr Comp Biol. 2013;53(2):359–72. 10.1093/icb/ict019. 23620251 10.1093/icb/ict019 PMC3710460

[17] Jin Z, Liu Y. DNA methylation in human diseases. Genes Dis. 2018;5(1):1–8. 10.1016/j.gendis.2018.01.002. 30258928 10.1016/j.gendis.2018.01.002 PMC6147084

[18] Zhong J, Agha G, Baccarelli AA. The role of DNA methylation in cardiovascular risk and disease: methodological aspects, study design, and data analysis for epidemiological studies. Circ Res. 2016;118(1):119–31. 10.1161/CIRCRESAHA.115.305206. 26837743 10.1161/CIRCRESAHA.115.305206 PMC4743554

[19] Krolevets M, Cate VT, Prochaska JH, Schulz A, Rapp S, Tenzer S, et al. DNA methylation and cardiovascular disease in humans: a systematic review and database of known CpG methylation sites. Clin Epigenet. 2023;15(1):56. 10.1186/s13148-023-01468-y. 10.1186/s13148-023-01468-y PMC10061871 36991458

[20] Salameh Y, Bejaoui Y, El Hajj N. DNA methylation biomarkers in aging and age-related diseases. Front Genet. 2020;11:171. 10.3389/fgene.2020.00171. 32211026 10.3389/fgene.2020.00171 PMC7076122

[21] Younesian S, Yousefi AM, Momeny M, Ghaffari SH, Bashash D. The DNA methylation in neurological diseases. Cells. 2022;11(21):3439. 10.3390/cells11213439. 36359835 10.3390/cells11213439 PMC9657829

[22] Brown WM. Exercise-associated DNA methylation change in skeletal muscle and the importance of imprinted genes: a bioinformatics meta-analysis. Br J Sports Med. 2015;49(24):1567–78. 10.1136/bjsports-2014-094073. 25824446 10.1136/bjsports-2014-094073

[23] King-Himmelreich TS, Schramm S, Wolters MC, Schmetzer J, Möser CV, Knothe C, et al. The impact of endurance exercise on global and AMPK gene-specific DNA methylation. Biochem Biophys Res Commun. 2016;474(2):284–90. 10.1016/j.bbrc.2016.04.078. 27103439 10.1016/j.bbrc.2016.04.078

[24] Kanzleiter T, Jähnert M, Schulze G, Selbig J, Hallahan N, Schwenk RW, et al. Exercise training alters DNA methylation patterns in genes related to muscle growth and differentiation in mice. Am J Physiol Endocrinol Metab. 2015;308(10):912–20. 10.1152/ajpendo.00289.2014. 10.1152/ajpendo.00289.2014 25805191

[25] Boyne DJ, King WD, Brenner DR, McIntyre JB, Courneya KS, Friedenreich CM. Aerobic exercise and DNA methylation in postmenopausal women: an ancillary analysis of the Alberta Physical Activity and Breast Cancer Prevention (ALPHA) Trial. PLoS One. 2018;13(6): e0198641. 10.1371/journal.pone.0198641. 29953441 10.1371/journal.pone.0198641 PMC6023230

[26] Streese L, Khan AW, Deiseroth A, Hussain S, Suades R, Tiaden A, et al. High-intensity interval training modulates retinal microvascular phenotype and DNA methylation of p66Shc gene: a randomized controlled trial (EXAMIN AGE). Eur Heart J. 2020;41(15):1514–9. 10.1093/eurheartj/ehz196. 31323685 10.1093/eurheartj/ehz196

[27] Moher D, Liberati A, Tetzlaff J, Altman DG, Altman D, Antes G, et al. Preferred reporting items for systematic reviews and meta-analyses: the PRISMA statement. PLoS Med. 2009;21: e1000097. 10.1371/journal.pmed.1000097. 10.1371/journal.pmed.1000097 PMC2707599 19621072

[28] Methley AM, Campbell S, Chew-Graham C, McNally R, Cheraghi-Sohi S. PICO, PICOS and SPIDER: a comparison study of specificity and sensitivity in three search tools for qualitative systematic reviews. BMC Health Serv Res. 2014;21:579. 10.1186/s12913-014-0579-0. 10.1186/s12913-014-0579-0 PMC4310146 25413154

[29] Maher CG, Sherrington C, Herbert RD, Moseley AM, Elkins M. Reliability of the PEDro scale for rating quality of randomized controlled trials. Phys The. 2003;83:713–21. 10.1093/ptj/83.8.713. 10.1093/ptj/83.8.713 12882612

[30] Chelly A, Bouzid A, Neifar F, Kammoun I, Tekari A, Masmoudi S, et al. Effect of aerobic/strength training on RANKL gene DNA methylation levels. J Phys Act Health. 2023;20(10):900–8. 10.1123/jpah.2022-0245. 37295782 10.1123/jpah.2022-0245

[31] Willmer T, Oosthuizen A, Dias S, Mendham AE, Goedecke JH, Pheiffer C. A pilot investigation of genetic and epigenetic variation of FKBP5 and response to exercise intervention in African women with obesity. Sci Rep. 2022;12(1):11771. 10.1038/s41598-022-15678-6. 35817784 10.1038/s41598-022-15678-6 PMC9273786

[32] Butts B, Butler J, Dunbar SB, Corwin E, Gary RA. Effects of exercise on ASC methylation and IL-6 cytokines in heart failure. Med Sci Sports Exerc. 2018;50(9):1757–66. 10.1249/mss.0000000000001641. 29683921 10.1249/mss.0000000000001641 PMC6095733

[33] Dimauro I, Scalabrin M, Fantini C, Grazioli E, Beltran Valls MR, Mercatelli N, et al. Resistance training and redox homeostasis: correlation with age-associated genomic changes. Redox Biol. 2016;10:34–44. 10.1016/j.redox.2016.09.008. 27687219 10.1016/j.redox.2016.09.008 PMC5040637

[34] Bryan AD, Magnan RE, Hooper AE, Harlaar N, Hutchison KE. Physical activity and differential methylation of breast cancer genes assayed from saliva: a preliminary investigation. Ann Behav Med. 2013;45(1):89–98. 10.1007/s12160-012-9411-4. 23054940 10.1007/s12160-012-9411-4 PMC3548059

[35] Denham J, O’Brien BJ, Harvey JT, Charchar FJ. Genome-wide sperm DNA methylation changes after 3 months of exercise training in humans. Epigenomics. 2015;7(5):717–31. 10.2217/epi.15.29. 25864559 10.2217/epi.15.29

[36] Ngwa JS, Nwulia E, Ntekim O, Bedada FB, Kwabi-Addo B, Nadarajah S, et al. Aerobic exercise training-induced changes on DNA methylation in mild cognitively impaired elderly African Americans: gene, exercise, and memory study-GEMS-I. Front Mol Neurosci. 2022;14: 752403. 10.3389/fnmol.2021.752403. 35110995 10.3389/fnmol.2021.752403 PMC8802631

[37] Hwang SH, Kang DW, Lee MK, Byeon JY, Park H, Park DH, et al. Changes in DNA methylation after 6-week exercise training in colorectal cancer survivors: a preliminary study. Asia Pac J Clin Oncol. 2022;18(1):52–60. 10.1111/ajco.13482. 33052030 10.1111/ajco.13482

[38] Sellami M, Bragazzi N, Prince MS, Denham J, Elrayess M. Regular, intense exercise training as a healthy aging lifestyle strategy: preventing DNA damage, telomere shortening and adverse DNA methylation changes over a lifetime. Front Genet. 2021;12: 652497. 10.3389/fgene.2021.652497. 34421981 10.3389/fgene.2021.652497 PMC8379006

[39] Šestáková Š, Šálek C, Remešová H. DNA methylation validation methods: a coherent review with practical comparison. Biol Proced Online. 2019;21:19. 10.1186/2Fs12575-019-0107-z. 31582911 10.1186/2Fs12575-019-0107-z PMC6771119

